# Mechanisms of Reversible Transition in Emulsions Stabilized by Modified Nanocrystalline Cellulose

**DOI:** 10.3390/molecules31101589

**Published:** 2026-05-09

**Authors:** Fei Liu, Xiaqing Li, Zhaoxiang Zhang, Yongfei Li, Xuewu Wang, Shaocan Dong

**Affiliations:** 1 College of Petroleum Engineering, Shandong Institute of Petroleum and Chemical Technology, Dongying 257061, China; 2 Efficient Exploration and Development of Oil and Gas Reservoirs and the Integration of Geology and Engineering, Shandong Provincial Engineering Research Center, Dongying 257061, China; 3 Petroleum Engineering Technology Research Institute of Shengli Oilfield Company, SINOPEC, Dongying 257000, China; 4 Chemistry and Chemical Engineering, Xi’an Shiyou University, Xi’an 710065, China; 5 College of Pipeline and Civil Engineering, China University of Petroleum (East China), Qingdao 266580, China

**Keywords:** modified nanocrystalline cellulose reversible emulsifier, organic clay, calcium chloride, reversible emulsion

## Abstract

Reversible emulsion drilling fluids integrate the advantages of water-based and oil-based systems, offering solutions to critical challenges in shale oil and gas development. However, conventional reversible emulsions face limitations including poor stability, high cost, and material scarcity. This research introduces widely available, eco-friendly modified nanocrystalline cellulose (MNCC) as a sustainable alternative. While current reversible drilling fluids primarily depend on organoclays and adopt aqueous phases containing 20–25% CaCl_2_ for shale inhibition, pH-responsive MNCC was validated as an effective reversible emulsifier capable of stabilizing emulsions through 48 consecutive phase-inversion cycles. Enhanced emulsion stability was achieved with organoclay at an optimal dosage (≤2.5 g/100 mL), and a composite interfacial film superior to the film formed by pure MNCC was fabricated via the combination of organoclay and MNCC. Increasing the organoclay content elevated the acid requirements for phase inversion (due to its lipophilicity) but reduced the alkali needs. Finally, higher CaCl_2_ concentrations in the aqueous phase reduced the acid demand for inversion yet increased alkali consumption and diminished stability in both oil-in-water (O/W) and water-in-oil (W/O) emulsions. These effects are attributed to the dual role of CaCl_2_ in compressing the electrical double layer and modifying phase density differences, synergistically governing reversible inversion behavior. This research provides a foundation for applying nanocrystalline cellulose-stabilized reversible emulsion drilling fluids, offering practical solutions for efficient development of sensitive reservoirs like shale.

## 1. Introduction

Petroleum and natural gas play an irreplaceable role as highly efficient energy sources in the national economy [1]. In recent years, shale oil and gas development has gradually broken through to establish production capacity, becoming a new protagonist in alleviating resource succession pressures. China possesses abundant shale oil and gas geological resources, with proven shale oil reserves ranking among the top globally, and shale resources accounting for a large proportion of unconventional oil and gas potential reserves. At present, the large-scale industrial exploitation of domestic shale oil and gas is being steadily promoted, and the annual exploitation volume continues to rise, showing huge development potential and broad application prospects. Achieving a leap in shale oil and gas production in China at this stage requires innovative breakthroughs in key development technologies as support. The shale technology revolution in the U.S. has enabled it to achieve energy self-sufficiency and verified the crucial role of advanced drilling and development technologies in shale resource exploitation. Among the conventional drilling fluid systems, oil-based drilling fluids are more suitable for the development of shale reservoirs due to their excellent shale inhibition and lubrication properties compared to water-based drilling fluids [1,2]. However, they bring challenges such as difficult mud cake removal [3], low cement bonding strength during cementing [4], emulsion blockage in formations, and difficulties in handling oil-containing drill cuttings and drilling waste (2 × 10^6^ tons/year) [5,6,7]. Reversible emulsion drilling fluids combine the advantages of water-based and oil-based drilling fluids, enabling the efficient development of shale oil and gas reservoirs. Currently, most reversible emulsion drilling fluid systems are stabilized by surfactant-type emulsifiers, resulting in poor stability [8,9,10]. Pickering emulsions stabilized by solid particles offer advantages of high stability and low toxicity [11,12] and find widespread applications in industries such as food, pharmaceuticals, cosmetics, and petroleum extraction [13,14,15,16,17]. While modified nano silica-stabilized reversible emulsion drilling fluids exhibit good stability, they face issues related to limited sourcing and high costs [18].

Cellulose, the most abundant and highest-yielding renewable natural polymer material synthesized by plants through photosynthesis [19,20,21], forms a system with alternating crystalline and amorphous regions. By selectively removing the amorphous regions from cellulose while retaining the crystalline regions through physicochemical means, nanocrystalline cellulose can be obtained [22]. Nanocrystalline cellulose possesses unique properties derived from its nanoscale effects, maintaining advantages such as abundant sourcing from renewable resources, biodegradability, and low environmental and health risks. By leveraging the advantages of nanocrystalline cellulose, the analysis indicates that using MNCC as an emulsifier to stabilize reversible emulsion drilling fluid systems can effectively enhance the stability of these fluids. MNCC itself offers unique advantages of wide sourcing, renewability, good biocompatibility, and biodegradability, making it a relatively ideal solution as a reversible emulsifier.

The factors influencing the performance of stable reversible emulsions with nanocrystalline cellulose are not yet fully understood, especially when considering the primary use of reversible emulsions in drilling fluid systems, where organic clay is added to adjust the rheological and stability properties to improve the performance of carrying drill cuttings. Additionally, in reversible emulsion drilling fluid systems, a 20% to 25% calcium chloride solution is commonly used in the water phase (to inhibit shale swelling). Furthermore, when reversible emulsions are used in other areas of oil fields, contact with high mineralization water during on-site fluid mixing increases the water phase mineralization level of the reversible emulsion. For this paper, laboratory-prepared pH-responsive MNCC was used as a reversible emulsifier, and the effects of organic clay and water phase calcium chloride concentration on reversible emulsions were investigated, with related mechanisms analyzed. The research provides a theoretical foundation and scientific basis for the application of nanocrystalline cellulose-stabilized reversible emulsion drilling fluid systems, offering important practical significance and application prospects in addressing the challenges faced in the efficient development of sensitive oil reservoirs like shale reservoirs.

## 2. Results and Discussion

### 2.1. Electron Microscope Analysis of MNCC Type Reversible Emulsifier

MNCC was prepared by the optimized condition mentioned in Section 3.2. Scanning electron microscopy (SEM) was employed to characterize both native nano-microcrystalline cellulose (NCC) and its modified counterpart (MNCC). This analysis enabled the observation of changes in surface elemental distribution during the treatment process, as well as a comparative assessment of morphological and compositional modifications before and after functionalization.

**Figure 1:**
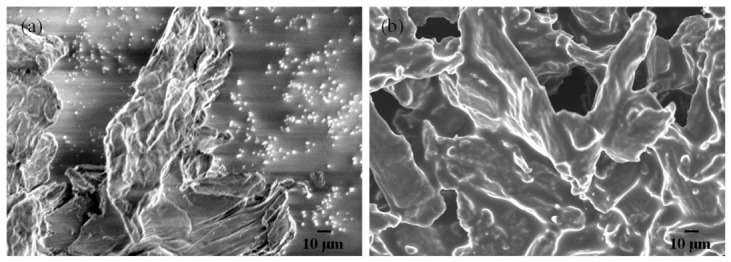
Electron microscope images of (**a**) nano-crystalline cellulose (NCC) and (**b**) modified nano-crystalline cellulose (MNCC).

Figure 1a displays the SEM image of nano-crystalline cellulose (NCC) before modification, revealing a fibrous and interconnected network structure with a smooth surface morphology. The image was acquired at an accelerating voltage of 5.00 kV and a working distance of 8.0 mm, with a scale bar of 10.0 μm indicating the micron-level dimensions of the aggregated NCC clusters. Figure 1b presents the SEM image of MNCC, still showing a relatively small particle size. Thus, the reversible emulsions stabilized by modified nano-crystalline cellulose (MNCC) exhibit enhanced structural stability at the oil–water interface. By comparing the stability of structures formed at the oil–water interface, the MNCC-based reversible emulsifiers form more robust structures than conventional surfactant-based systems. This is demonstrated by the significantly higher demulsification voltage required for MNCC-stabilized O/W emulsions (441 V) compared to their surfactant-stabilized counterparts (130 V). The 3.4-fold increase in critical demulsification voltage clearly indicates the superior interfacial stability imparted by MNCC-based emulsifiers.

**Figure 2:**
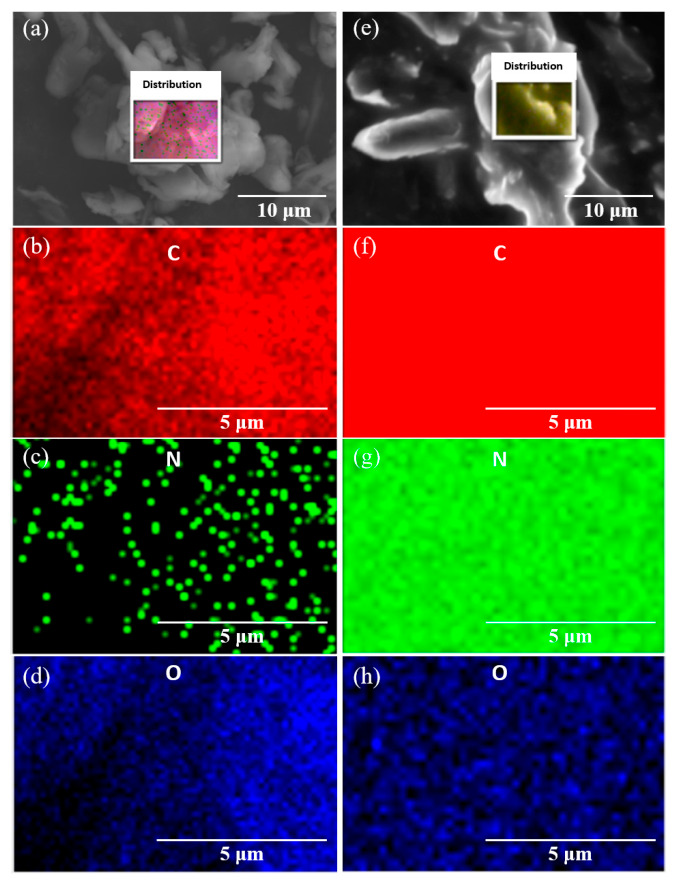
EDS stratified image for NCC and MNCC. (**a**,**e**) SEM morphology with overview elemental distribution; (**b**,**f**) carbon (C) mapping; (**c**,**g**) nitrogen (N) mapping; (**d**,**h**) oxygen (O) mapping.

A comparative analysis of energy-dispersive X-ray spectroscopy (EDS) was performed on NCC and MNCC under controlled experimental conditions (Figure 2). The EDS elemental mapping revealed a substantial increase in nitrogen content in MNCC compared to unmodified NCC. This observation is consistent with the anticipated chemical modification outcomes, confirming successful functionalization of the nanocellulose surface. The experimental results demonstrate good agreement with the designed modification protocol, validating the structural transformation from NCC to the target MNCC.

### 2.2. Performance of Reversible Emulsions Under Repeated Phase Inversion

Reversible emulsions offer significant advantages, not only by combining the beneficial properties of different liquid phases but also through their capacity to undergo multiple reversible phase transitions, thereby enhancing their reusability and environmental sustainability. The practical utility of such emulsions is largely determined by the number of reversible phase transitions they can achieve, which directly correlates with their potential for repeated use. However, this requires the reversible emulsifier to exhibit high shear resistance and the system to maintain stability across varying oil–water ratios.

In this research, the multiple reversible phase transition behavior of an emulsion stabilized by emulsifier was systematically evaluated. A 200 mL reversible emulsion was prepared and subjected to pH-triggered phase inversion experiments. The acid-induced transition point was set at pH 6.0, ensuring near-complete conversion of the reversible emulsifier into anionic surfactants, while the alkali-induced transition point was set at pH 8.5, facilitating its transformation into non-ionic emulsifiers. These conditions were optimized to maximize phase reversibility and assess the emulsion’s stability under cyclic transitions. This investigation provides critical insights into the design of robust reversible emulsions, highlighting the importance of pH-responsive emulsifiers in achieving controlled and repeatable phase inversion for sustainable applications.

**Figure 3:**
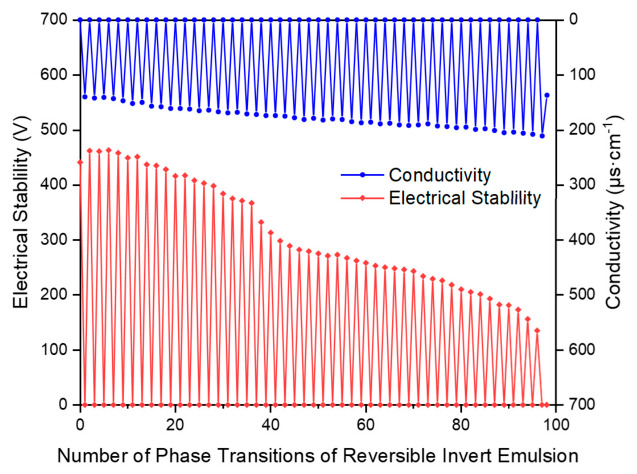
Reversible emulsion repeated reversible phase performance test.

The experimental results (Figure 3) demonstrate that the reversible emulsion stabilized by emulsifier exhibits exceptional phase transition stability, achieving 97 phase transitions, equivalent to 48 fully reversible cycles. This performance significantly surpasses that of conventional reversible emulsion systems, including the Shengli Oilfield reversible emulsion system (10 reversible cycles) and the laboratory-synthesized DMOB reversible emulsion drilling fluid system (32 reversible cycles). The marked improvement in reversibility indicates that the emulsifier possesses high shear resistance and maintains structural integrity under repeated phase inversion. Furthermore, the emulsion demonstrates robust adaptability to varying oil–water ratios, confirming its superior reversible phase stability and potential for long-term reuse in practical applications.

### 2.3. Mechanism of Organic Clay Effects on Reversible-Emulsion

Organic clay is prepared by replacing the cations adsorbed between montmorillonite layers with organic cations. It exhibits excellent thickening, thixotropic properties, suspension stability, and high-temperature stability. These characteristics have led to widespread industrial applications in coating formulations, printing inks, petroleum engineering, and environmental remediation processes [23,24,25]. Given the primary use of reversible emulsions, organic clay is added to the system to adjust its rheological and stabilizing properties [26,27,28,29], thereby enhancing the performance of the reverse emulsion drilling fluid in carrying cuttings. Therefore, studying the impact of organic clay on reversible emulsions can facilitate the promotion and application of self-made reversible emulsion systems in oil fields.

A comprehensive evaluation of phase reversal behavior in emulsions prepared with varying organic clay concentrations was conducted. Our experimental results established a critical threshold for organic clay incorporation, demonstrating that reversible emulsion formation is achievable at organic clay concentrations ≤ 2.5 g/100 mL (Table 1). This finding delineates the operational parameters for effective utilization of organic clay in reversible emulsion systems.

Within the established effective range of organic clay incorporation (0–2.5 g/100 mL), four experimental groups were systematically evaluated: 0, 1.0, 2.0, and 2.5 g/100 mL organic clay concentrations. Changes in emulsion properties (static stability, demulsification voltage, and electrical conductivity) with increasing hydrochloric acid (HCl) dosage under different organic clay conditions were investigated. Comparative analysis of these parameters enabled rigorous assessment of organoclay’s influence on reversible emulsion performance.

Given the pH-dependent nature of emulsion phase reversal, preliminary evaluation of organic clay’s acid/alkali consumption behavior was essential. Specifically, whether organic clay can sequester H^+^/OH^−^ ions to potentially interfere with the phase transition mechanism was examined. The organic clay dispersion protocol involved the following: (1) gradual addition of organic clay to the liquid medium under continuous stirring, followed by (2) homogenization at 300 rpm for 5 min in a sealed container, before (3) immediate characterization of the resulting dispersion.

**Figure 4:**
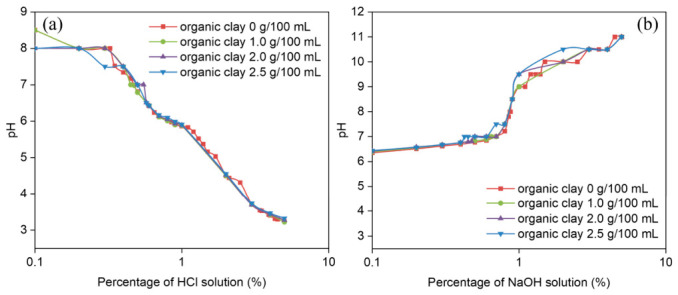
pH values of (**a**) HCl and (**b**) NaOH added in the reversible emulsion system with different dosages of organic clay.

It was demonstrated that varying dosages of organic clay exhibit negligible influence on the pH of both acidic and alkaline systems (Table 2). This observation indicated that the organic clay used in this research did not significantly sequester H^+^ or OH^−^ ions under acidic or alkaline conditions (Figure 4). Furthermore, structural analysis of the organic clay confirmed that it did not consume H^+^/OH^−^ ions [30]. Consequently, when investigating the effects of organic clay on the phase transition behavior of reversible emulsions, the potential consumption of H^+^ or OH^−^ by the organoclay can be disregarded.

#### 2.3.1. Acid-Induced Phase Transition Mechanism of Reversible Emulsions by Organic Clay

**Figure 5:**
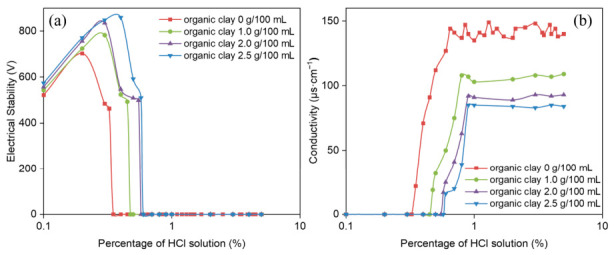
Influence of HCl on (**a**) demulsification voltage and (**b**) conductivity during acid-induced phase transition of reversible emulsions at different organic clay additions.

The influence of organic clay on the demulsification voltage and conductivity of reversible emulsions was systematically investigated as a function of incremental HCl addition (Figure 5). These experimental measurements enabled a quantitative analysis of how the acid-induced phase transition point shifts with increasing organic clay addition.

The pH values of acid-induced phase inversion for emulsion systems with different organic clay contents are as follows: the phase inversion pH is 7.52 at 0% organic clay, 6.98 at 1.0% organic clay, 6.51 at 2.0% organic clay, and 6.43 at 2.5% organic clay. Notably, all prepared water-in-oil emulsions maintained excellent electrical stability (electrical stability exceeding 200 V) throughout the entire stable water-in-oil emulsion stage. The acid-induced phase transition points of reversible emulsions under different organic clay addition conditions are shown in Figure 5, including ① HCl (5%) at 0.35% (organic clay 0 g/100 mL); ② HCl (5%) at 0.475% (organic clay 1.0 g/100 mL); ③ HCl (5.0%) at 0.575% (organic clay 2.0 g/100 mL); ④ HCl (5.0%) at 0.60% (organic clay 2.5 g/100 mL). This phenomenon can be attributed to the formation of an organic clay–MNCC composite emulsifier film at the oil–water interface upon organic clay incorporation into the emulsion system. As the organic clay concentration increases, its relative proportion within the composite interfacial film rises correspondingly. Given the inherent hydrophobicity of organic clay, the composite film exhibits progressively greater hydrophobic character with higher organoclay content. This led to an increase in the acid-induced phase transition point of the reversible emulsion with higher organic clay addition. In other words, the addition of organic clay makes it more difficult for the reversible emulsion to achieve acid-induced phase transition.

**Figure 6:**
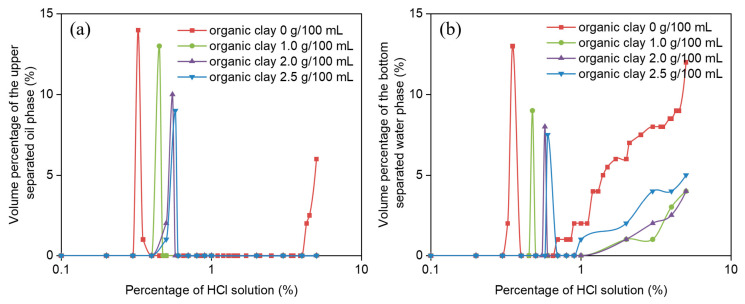
Influence of HCl on (**a**) oil separation rate and (**b**) water separation rate during acid-induced phase transition of reversible emulsions at different organic clay additions.

The variations of water separation rate and oil separation rate during the acid-induced phase transition of reversible emulsion under different organic soil dosages with the increase of hydrochloric acid dosage are shown in Figure 6. Since there are few groups of the O/W emulsion stage in the reversible emulsion acid-induced phase transition process, and it includes the O/W emulsion phase transition stage, the stability of the O/W emulsion with the addition of organic clay is mainly compared in this process. In the W/O emulsion stage, after 24 h of static settling, the water and oil phase separation rates decrease with increasing organic clay addition (Figure 6a). This is because organic clay forms a composite emulsifier film with MNCC at the oil–water interface. As the amount of organic clay increases, the proportion of organic clay in the composite emulsifier film increases, enhancing the stability of the composite emulsifier film, thus increasing the stability of the W/O emulsion. Therefore, as the organic clay loading increases, the difficulty of oil droplet disruption increases, leading to a decrease in oil phase separation rates. Additionally, during the 24 h settling period, the amount of oil droplets disrupted and separated decreases with increasing organic clay loading. Thus, as the organic clay concentration increases, the volume of the oil phase in the W/O emulsion increases, resulting in a reduction in the water phase separation rates.

Under different organic clay addition conditions, the water and oil precipitation rates at the acid-induced phase transition point of reversible emulsions are both high, and the oil precipitation rate decreases with increasing organic clay addition (Figure 6b). The phenomena can be attributed to two primary factors: (1) the reversible emulsion has just completed the acid-induced phase transition, and the oil–water interface emulsifier (a composite emulsifier of emulsifier and organic clay) cannot stabilize the O/W emulsion very well. At this time, the stability of the O/W emulsion is relatively poor, so the water separation rate and oil separation rate are relatively high after standing for 24 h. (2) As the amount of organic clay increases, the proportion of organic clay at the oil–water interface increases, leading to an enhanced stability of the reversible emulsion at the acid-induced phase inversion point with the increase of organic clay dosage. Therefore, with the increase of the organic clay dosage, fewer oil droplets are broken and separated within the 24 h static time, and the oil separation rate decreases with the increase of the organic clay dosage.

#### 2.3.2. Alkali-Induced Phase Transition Mechanism of Reversible Emulsions by Organic Clay

**Figure 7:**
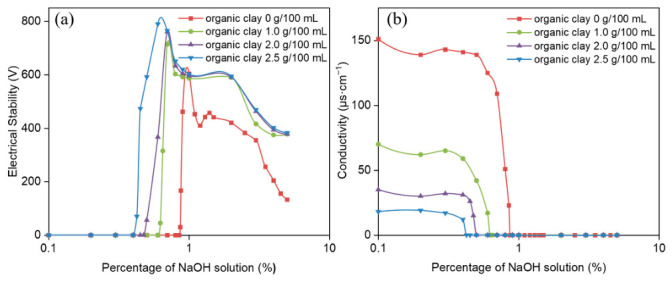
Influence of NaOH solution on (**a**) demulsification voltage and (**b**) conductivity during alkali-induced phase transition of reversible emulsions at different organic clay additions.

The influence of organic clay on the demulsification voltage and conductivity of reversible emulsions was systematically investigated as a function of incremental NaOH addition (Figure 7). These experimental measurements enabled a quantitative analysis of how the alkali-induced phase transition point shifts with the increasing addition of organic clay. The alkali-induced phase inversion pH values of emulsions with varying organoclay dosages were determined as 8.0 (0% organoclay) and 7.0 (1.0%, 2.0%, 2.5% organoclay), respectively. Moreover, the pH ranges for emulsions with high electrical stability (>200 V) after alkali-induced phase inversion were clarified. The stable pH range was 8.5–10.5 for the emulsion with 0% organoclay, while emulsions supplemented with 1.0%, 2.0%, and 2.5% organoclay maintained superior electrical stability at pH values above 7.0.

The alkali-induced phase transition points of reversible emulsions under different organic clay addition conditions are shown in Figure 7, including ① NaOH solution (5.0%) at an addition rate of 0.87% (organic clay 0 g/100 mL); ② NaOH (5.0%) at an addition rate of 0.625% (organic clay 1.0 g/100 mL); ③ NaOH solution (5.0%) at an addition rate of 0.50% (organic clay 2.0 g/100 mL); ④ NaOH solution (5.0%) at an addition rate of 0.425% (organic clay 2.5 g/100 mL). The initial W/O emulsion was prepared with HCl (5.00%) at an addition rate of 0.65%. This result can be attributed to the composite emulsifier film formed between organic clay and MNCC at the oil–water interface. The proportion of organic clay in the composite emulsifier film increases with the increase in organic clay addition. Organic clay exhibits hydrophobicity, so the composite emulsifier film shows stronger hydrophobicity as the proportion of organic clay increases, leading to a decrease in the alkaline phase transition point of the reversible emulsion with increasing organic clay addition. This means that the addition of organic clay makes it easier for the reversible emulsion to achieve an alkaline phase transition. At the same time, the overall droplet size of the W/O emulsion increases with the increase in organic clay addition, reducing the amount of alkali solution required for the alkaline phase transition of the reversible emulsion, thus decreasing the difficulty of the alkaline phase transition.

**Figure 8:**
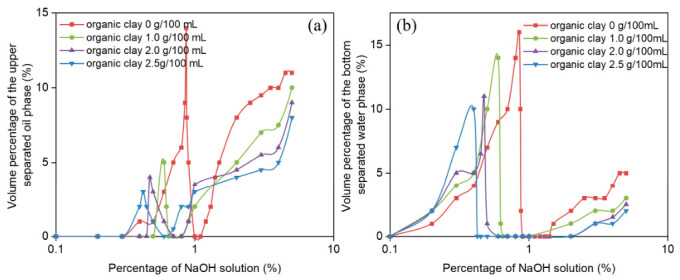
Effect of NaOH solution on (**a**) oil precipitation rate and (**b**) water precipitation rate during alkali-induced phase transition of reversible emulsions at different organic clay additions.

The water and oil separation rates during the reversible emulsion phase transition of different organic clay additions (Figure 8) were compared as the NaOH solution increased. Since there are fewer stages in the reversible emulsion phase transition process, and these include the phase transition stage of W/O emulsions, the main focus was on comparing the stability of the O/W emulsions with the changes in organic clay addition. In the O/W emulsion stage, after 24 h of static observation, the water and oil separation rates decreased as the organic clay addition increases. The primary reason is that under conditions where organic clay is added to the emulsion system, a composite emulsifier film formed by organic clay and MNCC is created. The proportion of organic clay in this composite emulsifier film increases with the addition of organic clay, and the stability of the composite emulsifier film is enhanced as the proportion of organic clay increases. Therefore, the stability of the O/W emulsion also increases with the addition of organic clay. Additionally, due to the increased structural density between water droplets as the organic clay addition increases, water droplets are less likely to be disrupted and separated, leading to a decrease in the water separation rate. This is also because the structural density between water droplets increases with the addition of organic clay, making it harder for the structures to be disrupted and preventing water droplets from becoming tightly packed. Consequently, the oil separation rate of the O/W emulsion decreases as the addition of organic clay increases.

The water and oil precipitation rates at the reversible emulsion alkaline phase transition point are both high, and the water precipitation rate decreases with increasing organic clay (Figure 8). This can be explained in that the reversible emulsion has just completed its alkaline phase transition. The emulsifier at the oil–water interface (emulsifier and the composite emulsifier of organic clay) cannot effectively stabilize the O/W emulsion, meaning that the stability of the O/W emulsion is poor. Therefore, after 24 h of settling, the water and oil precipitation rates are higher. Additionally, as the organic clay addition increases, the proportion of organic clay at the oil–water interface also increases, leading to enhanced stability of the emulsion at the reversible emulsion alkaline phase transition point with increasing organic clay addition. Thus, as the organic clay addition increases, fewer water droplets break out during the 24 h settling period, resulting in a decrease in the water precipitation rate with increasing organic clay.

In the context of emulsion systems, organic clay and MNCC combine to form a composite emulsifier film at the oil–water interface. Within this composite film, the proportion of organic clay rises in tandem with an increase in the amount of organic clay added. Given that the stability of the composite emulsifier film surpasses that of a film composed solely of MNCC, the stability of both O/W emulsions and W/O emulsions is enhanced with the augmentation of organic clay dosage. Furthermore, due to the lipophilic (oil-affinitive) nature of organic clay, the structural compactness between water droplets in W/O emulsions intensifies as the dosage of organic clay increases. Consequently, the quantity of acid required to induce acid-triggered phase inversion in reversible emulsions escalates in correlation with the rising amount of organic clay. On the other hand, in O/W emulsions, the overall droplet size expands with the addition of organic clay, while the uniformity of droplet size distribution deteriorates as the dosage of organic clay goes up. These morphological alterations facilitate alkali-induced phase inversion in reversible emulsions, rendering the process less challenging. As a result, both the difficulty level and the requisite amount of alkali for alkali-triggered phase inversion in reversible emulsions diminish with an increase in the dosage of organic clay.

### 2.4. Mechanism of Aqueous CaCl_2_ Concentration Impacting Reversible Emulsion

The principal application of reversible emulsions in petroleum operations resides in reversible emulsion drilling fluids. These systems conventionally employ aqueous phases comprising 20–25% CaCl_2_ solution. High-concentration CaCl_2_ solutions function as an efficacious shale inhibitor, suppressing clay hydration swelling and dispersion through double-layer compression on the clay surfaces. This mechanism reduces the hydration film thickness, diminishes the zeta potential, and enhances the shale stability [31,32,33]. When reversible emulsions are used in other areas of oil fields, they inevitably experience interfacial exposure to high-salinity formation waters. This interaction elevates the mineralization degree of the aqueous phase of the reversible emulsion. Given the heterogeneity of the formation conditions and the variable make-up water compositions, aqueous phase mineralization exhibits significant spatial and temporal variation. Consequently, investigations into salinity impacts on reversible emulsion behavior must be targeted at specific formation conditions. Thus, the influence of CaCl_2_ concentration in the aqueous phase on reversible emulsion phase inversion phenomena was investigated, with a reference provided for research on the impact of aqueous phase mineralization on reversible emulsions. Clarifying the influence of aqueous phase CaCl_2_ concentration on reversible emulsions also aids in the promotion and application of self-made reversible emulsion systems in oil fields. The viability of preparing reversible emulsions in aqueous phases containing 0–30 wt% CaCl_2_ was systematically evaluated. Preliminary analysis established that stable oil-in-water (O/W) emulsions could be reproducibly formulated at aqueous CaCl_2_ concentrations ≤30 wt%. Experimental validation confirmed reversible emulsion formation at three strategic CaCl_2_ concentrations: 0 wt%, 15 wt%, and 30 wt% (Table 3). The dosage of organic clay was 0, and the addition amount of MNCC was 1.60 g/100.00 mL oil phase. For each system, we quantitatively investigated the evolution of the emulsion properties, including the electrical stability, demulsification voltage, and conductivity, as functions of HCl dosage. A comparative analysis was subsequently performed to elucidate the concentration-dependent influence of aqueous CaCl_2_ on emulsion phase behavior and property modulation.

#### 2.4.1. CaCl_2_ Concentration Effects on Acid-Induced Phase Transition in Reversible Emulsions

**Figure 9:**
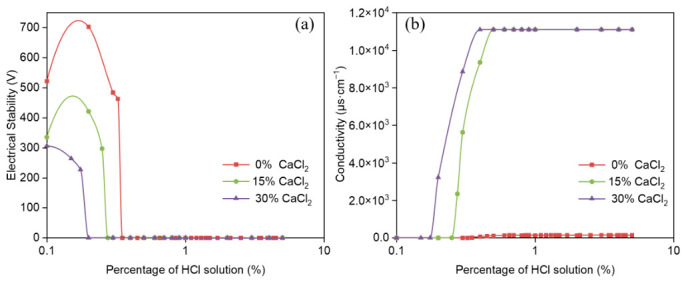
HCl impact on (**a**) demulsification voltage and (**b**) conductivity during reversible emulsion acid phase transition across aqueous CaCl_2_ concentrations.

The fundamental properties of initial O/W emulsions formulated with varying CaCl_2_ aqueous phase concentrations, including: demulsification voltage, conductivity, oil precipitation rate (24 h), water precipitation rate (24 h) are summarized in Table 4. The demulsification voltage and conductivity of the emulsion prepared under different CaCl_2_ concentrations in the water phase were studied with the increase of HCl addition (Figure 9). The acid-induced phase transition point of the reversible emulsion was analyzed with the increase of CaCl_2_ concentration in the water phase.

**Figure 10:**
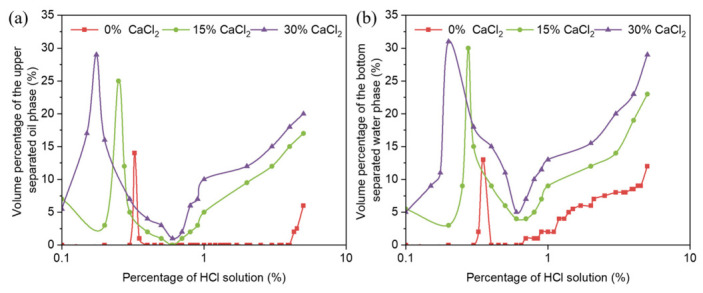
HCl impact on the oil (**a**) and water (**b**) separation rate during acid-induced phase transition across aqueous CaCl_2_ concentrations.

Regarding emulsion systems with different calcium chloride concentrations in the aqueous phase, the acid-induced phase inversion occurred at pH 7.52 (0% CaCl_2_), pH 7.90 (15% CaCl_2_), and pH 7.90 (30% CaCl_2_). Similarly, all water-in-oil emulsions formed in the corresponding pH range possessed favorable electrical stability, with the electrical stability value consistently higher than 200 V. Figure 9 reveals distinct acid-induced phase transition points for reversible emulsions across varying aqueous CaCl_2_ concentrations: ① HCl (5%) with an addition of 0.35% (water phase CaCl_2_ 0 wt%); ② HCl (5%) with an addition of 0.28% (water phase CaCl_2_ 15.00 wt%); ③ HCl (5%) with an addition of 0.2% (water phase CaCl_2_ 30 wt%). Further analysis was conducted based on the stability of the emulsion during static settling and microscopic structure. The static stability of emulsions undergoing acid-induced phase transition under different aqueous CaCl_2_ concentrations was characterized. Figure 10 presents the oil/water precipitation rates during acid-induced phase inversion of reversible emulsions across varying aqueous CaCl_2_ concentrations. Given that the acid-induced phase inversion process involves fewer stages for O/W emulsions—including the phase inversion stage itself—this study primarily compared the stability of O/W emulsions as the CaCl_2_ concentration in the aqueous phase varied. During the O/W emulsion stage, under constant HCl addition, static observation for 24 h revealed that both the water and oil precipitation rates increased with higher CaCl_2_ concentrations in the aqueous phase.

The oil precipitation rate increased with higher CaCl_2_ concentration in the aqueous phase. This occurs primarily because the density of the aqueous phase increases with rising CaCl_2_ concentration, enlarging the density difference between the aqueous and oil phases. Consequently, the buoyancy of the oil droplets increases. Additionally, inorganic salts exert a shielding effect, reducing electrostatic repulsion between the water droplets as the CaCl_2_ concentration rises, thereby promoting water droplet coalescence. During this process, the overall size of the oil droplets within the W/O emulsion increases, while their size uniformity decreases. This signifies reduced stability of the oil droplets. Therefore, under constant HCl dosage, the 24 h oil precipitation rate of the W/O emulsion increases with higher aqueous phase CaCl_2_ concentration.

The water separation rate also increases with higher aqueous phase CaCl_2_ concentration. Enhanced buoyancy of oil droplets in the O/W emulsion facilitates their tighter packing. Furthermore, increased CaCl_2_ concentration promotes greater disruption of the oil droplets, leading to a reduced oil phase volume within the O/W emulsion. Thus, under identical HCl addition conditions, the 24 h water separation rate of the O/W emulsion increases with rising CaCl_2_ concentration.

At the acid-induced phase transition point of the reversible emulsion, both the water yield and oil yield are high across the varying aqueous phase CaCl_2_ concentrations, with the oil yield increasing as the CaCl_2_ concentration rises (Figure 10). This is primarily due to two factors: (a) The reversible emulsion has just undergone phase inversion. At this point, the emulsifier at the oil–water interface is ineffective at stabilizing the newly formed O/W emulsion, resulting in inherently poor stability and consequently higher water and oil yields after 24 h of settling. (b) Higher aqueous CaCl_2_ concentration weakens the electrostatic repulsion between the oil droplets and increases the oil–water density difference. This facilitates the formation of dense oil droplet clusters within the O/W emulsion. Consequently, the stability of the emulsion at the phase transition point decreases as the CaCl_2_ concentration increases. Therefore, during the 24 h settling period, more oil droplets break out, causing the oil yield to rise with higher aqueous CaCl_2_ concentration.

#### 2.4.2. CaCl_2_ Concentration Effects on Alkali-Induced Phase Transition in Reversible Emulsions

The effect of CaCl_2_ on the reversible emulsion alkali-induced phase transition should first consider the influence of calcium ion and OH^−^ reaction to form calcium hydroxide precipitate in the CaCl_2_ solution. The precipitation equilibrium constant (solubility product) Ksp of Ca(OH)_2_ precipitate is 5.36 × 10^−5^, Equation (1) is used for the calculation [34,35]:

Ksp (Ca (OH)_2_) = [Ca^2+^] [OH^−^]^2^ (1)

The density of a 15 wt% CaCl_2_ aqueous solution is 1.13 g/cm^3^. At this concentration, the molar concentration of Ca^2+^ is 1.53 mol/L, corresponding to a saturated OH^−^ concentration of 5.93 × 10^−3^ mol/L. This OH^−^ concentration equates to pH 11.77. Therefore, in a 15 wt% CaCl_2_ solution, Ca(OH)_2_ precipitation occurs when the aqueous phase pH exceeds 11.77.

The density of a 30 wt% CaCl_2_ aqueous solution is 1.29 g/cm^3^. Given this concentration, the molar concentration of Ca^2+^ is 3.48 mol/L. The corresponding saturated molar concentration of OH^−^ is 3.93 × 10^−3^ mol/L, equivalent to a pH of 11.60. Therefore, in a 30 wt% CaCl_2_ solution, Ca(OH)_2_ precipitation occurs when the aqueous phase pH exceeds 11.60.

Considering that a reversible emulsion with 0 wt% CaCl_2_ addition reaches pH 11.00 during acid-induced phase inversion when 5% HCl (5 wt%) is added, this value is below the pH required for Ca(OH)_2_ precipitation in both 15 wt% (pH 11.77) and 30 wt% (pH 11.60) CaCl_2_ aqueous solutions. Therefore, Ca(OH)_2_ precipitation effects can be neglected during the alkaline-induced phase inversion of the reversible emulsion.

**Figure 11:**
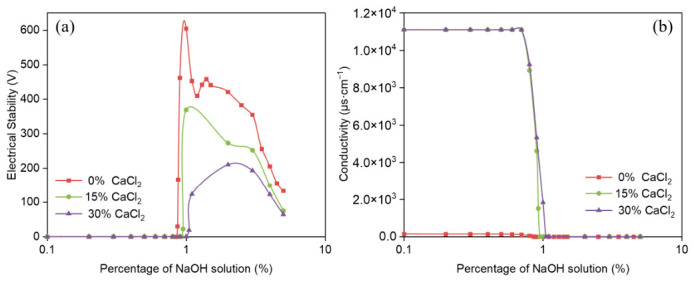
NaOH impact on (**a**) demulsification voltage and (**b**) conductivity during reversible emulsion phase transition across aqueous CaCl_2_ concentrations.

Preliminary tests were conducted on the basic properties of the initial W/O emulsion prepared using different concentrations of CaCl_2_ solution. These properties included the demulsification voltage, conductivity, 24 h oil precipitation rate, and 24 h water precipitation rate (Table 5). Figure 11 shows the trends in the demulsification voltage and conductivity with increasing NaOH solution (5.00 wt%) addition for reversible emulsions containing different aqueous phase CaCl_2_ concentrations. The alkaline demulsification points occur at the following NaOH additions: ① 0.87% NaOH solution (water phase CaCl_2_ concentration 0.00 wt%); ② 0.95% NaOH solution (water phase CaCl_2_ concentration 15.00 wt%); ③ 1.05% NaOH solution (water phase CaCl_2_ concentration 30.00 wt%).

**Figure 12:**
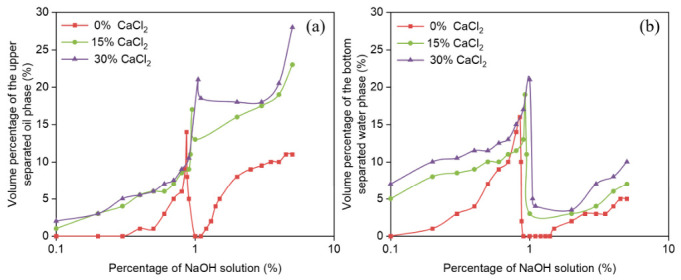
Impact of NaOH solution on (**a**) oil and (**b**) water precipitation rates during alkaline phase transition across aqueous CaCl_2_ concentrations.

Comparative analysis of the water and oil precipitation rates during the reversible emulsion phase transition with NaOH solution (Figure 12) reveals minimal presence of W/O emulsion stages—including transitions between W/O states. The analysis therefore focused on O/W emulsion stability across aqueous phase CaCl_2_ concentrations. In this O/W emulsion stage, both precipitation rates increase with higher CaCl_2_ concentration after 24 h settling. This trend can be primarily attributed to the following reasons. (1) The water separation rate increases with higher aqueous phase CaCl_2_ concentration. This occurs primarily because increased CaCl_2_ concentration raises the aqueous phase density, thereby enlarging the oil–water density difference. This enhanced density difference promotes water droplet coalescence. Consequently, overall droplet size increases within the O/W emulsion and the uniformity of droplet size decreases, indicating reduced stability of the water droplets. Therefore, under identical HCl addition, the 24 h water separation rate of the O/W emulsion increases with higher aqueous phase CaCl_2_ concentration. (2) The oil precipitation rate rises with increasing aqueous phase CaCl_2_ concentration. The enlarged oil–water density difference facilitates tighter packing of the water droplets. Additionally, the water phase volume within the oil–water emulsion decreases at higher CaCl_2_ concentrations. Both factors contribute to increased oil precipitation rates in the emulsion as the aqueous CaCl_2_ concentration rises. (3) At the reversible emulsion’s alkaline inversion point, both the water and oil precipitation rates are high across all CaCl_2_ concentrations, with the water precipitation rate increasing at higher concentrations (Figure 12). This results from the following. (a) Emulsifier failure: immediately post-inversion, the emulsifier cannot stabilize the new O/W emulsion, causing inherent instability and high separation rates after 24 h settling. (b) Enhanced separation dynamics: higher aqueous CaCl_2_ concentrations increase the oil–water density differences, promoting water droplet accumulation and coalescence in the O/W emulsion. This reduces emulsion stability at the inversion point. Consequently, more water droplets separate during the 24 h settling as the CaCl_2_ concentration increases, elevating the water precipitation rates.

These results demonstrate an inverse relationship between aqueous phase CaCl_2_ concentration and the acid/alkali required for reversible emulsion phase transition. For acid-induced transition, the acid requirement decreases with higher aqueous CaCl_2_ concentration. For alkali-induced transition, the NaOH requirement increases with higher aqueous CaCl_2_ concentration. Additionally, these results indicate how these transition points shift with aqueous CaCl_2_ concentration. The stability and phase inversion of reversible emulsions depend critically on the distribution of different emulsifiers at the oil–water interface. Since the zeta potential of O/W emulsions characterizes this emulsifier distribution, the variation in zeta potential of reversible emulsions (as O/W emulsions) with aqueous phase CaCl_2_ concentration was first measured.

Reversible emulsion HCl (5.00%) was added to 0.65% to prepare O/W emulsion and its zeta potential was tested. Due to poor light transmittance, the emulsion was diluted (0.05%) before the zeta potential test.

**Figure 13:**
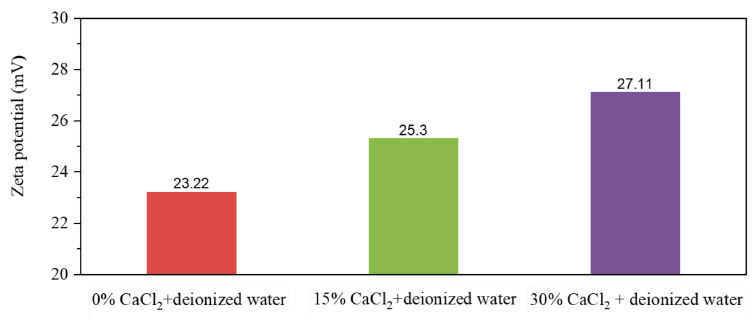
Zeta potential of different types of emulsions diluted with deionized water.

Emulsions prepared from varying CaCl_2_ concentrations and diluted with deionized water (Figure 13) demonstrate that aqueous CaCl_2_ concentration modulates the emulsifier composition on oil droplet surfaces in W/O emulsions. Specifically, the oil droplet surface charge increases with higher CaCl_2_ concentrations. Microscopy reveals minimal CaCl_2_ concentration impact on the emulsion particle volume during initial phase inversion. These observations indicate enhanced proportions of ionic emulsifiers on oil droplet surfaces at elevated aqueous CaCl_2_ concentrations. This effect primarily stems from electrostatic shielding by high-concentration salt solutions [36,37,38], which reduces inter-emulsifier repulsion and facilitates ionic MNCC distribution at the oil–water interface.

Increasing the aqueous phase CaCl_2_ concentration not only enhances the proportion of ionic emulsifiers on the emulsion droplet surfaces but also induces electrical double layer compression. To mitigate the confounding effects of variable CaCl_2_ concentrations on zeta potential measurements, we employed high-ratio dilution with deionized water for all O/W emulsions. This approach partially eliminates ionic strength-dependent electrostatic screening, enabling more accurate characterization of the intrinsic oil–water interface electrostatics.

**Figure 14:**
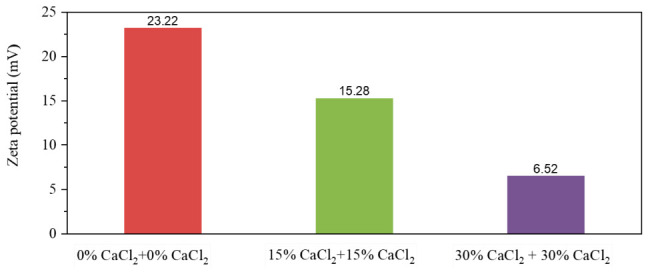
Zeta potential of different types of emulsion diluted CaCl_2_ aqueous solution.

For comparative analysis, all O/W emulsions—originally prepared with different CaCl_2_ concentration aqueous solutions—were diluted using their respective CaCl_2_ concentration solutions prior to measurement. The experimental results are shown in Figure 14. For W/O emulsions, the electrical double layer compression induced by high-concentration CaCl_2_ solutions dominates over the effect of the increased average MNCC charge on the droplet surfaces. Consequently, the emulsion zeta potential decreases significantly with rising CaCl_2_ concentration. This indicates reduced electrostatic repulsion between the oil droplets in W/O emulsions at higher CaCl_2_ concentrations—a key factor explaining the decreasing W/O emulsion stability with increasing CaCl_2_. Combining this interfacial MNCC distribution analysis with reversible emulsion transition points (acid-induced at 0.65% HCl (5.00%), alkali-induced as previously determined), Table 6 comprehensively summarizes the aqueous phase CaCl_2_ concentration effects.

To further rigorously verify the mechanism of microscopic instability induced by CaCl_2_, we conducted an in-depth quantitative analysis combined with the zeta potential test results. The introduction of high-concentration CaCl_2_ solutions (such as 15 wt% and 30 wt%) not only significantly altered the density of the aqueous phase and widened the macroscopic density difference between oil and water but, more importantly, the high ionic strength it brought about triggered a strong electrostatic shielding effect. The zeta potential data in Figure 14 directly and quantitatively indicate that with the increase of CaCl_2_ concentration in the aqueous phase, the electric double layer compression effect caused by high salt concentration became dominant, resulting in a remarkable decrease in the absolute value of the emulsion’s zeta potential.

This quantitative variation in interfacial charge characteristics directly confirms that the electrostatic repulsion between the droplets is greatly weakened. At the micromechanical level, when the continuously declining electrostatic repulsion can no longer effectively resist the flocculation and coalescence tendency driven by the gradually increasing macroscopic density difference, the emulsion will inevitably suffer from macroscopic instability, manifested as increased water separation rate and oil separation rate as well as decreased demulsification voltage. The above findings not only convert the original hypothesis of “electric double layer compression” into quantitative evidence directly supported by zeta potential data but also establish a clear and rigorous closed physicochemical correlation among ionic strength (CaCl_2_ concentration), microscopic interfacial behavior (zeta potential attenuation), and macroscopic emulsion stability (phase separation and reduced electrical stability).

**Figure 15:**
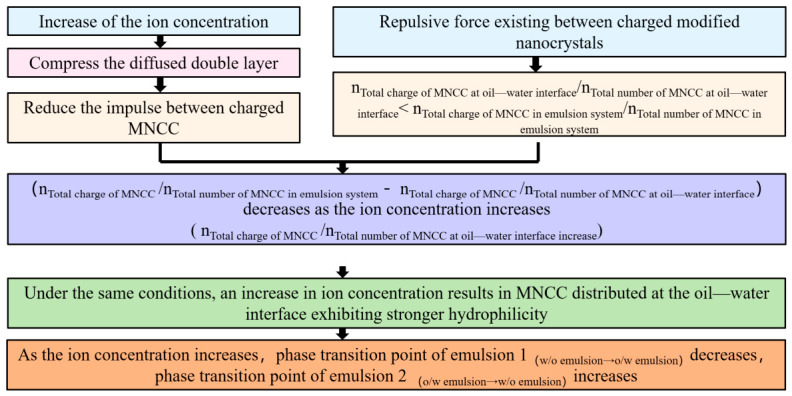
Effect of aqueous CaCl_2_ concentration on reversible emulsion properties.

For reversible emulsion phase transition points, the effect of increased MNCC particle charge at the droplet surfaces (caused by electrical double layer compression from high-concentration CaCl_2_ solutions) dominates over the effect of reduced emulsifier hydrophilicity (similarly caused by this compression). Consequently, the acid dosage required for acid-induced phase transition decreases with rising CaCl_2_ concentration. The alkali dosage required for alkali-induced phase transition increases with rising CaCl_2_ concentration (Figure 15).

## 3. Materials and Methods

### 3.1. Materials

Hydrochloric acid, sodium hydroxide, calcium chloride, and nanocrystalline cellulose CNC-4 were purchased from Sinopharm Chemical Reagent Co., Ltd.(Shanghai, China). MNCC was lab-made; 5# white oil is supplied by the French Scaran Group; The organic clay was provided by Shengli Oilfield Drilling Engineering Technology Co., Ltd. (Dongying, China).

### 3.2. Preparation and Characterization of MNCC

Add 10 g of nanocrystalline cellulose into a 250 mL vacuum flask. Evacuate the flask using a vacuum pump for 3 h. While maintaining the vacuum, introduce 30 g of methyltrimethoxysilane (less than 1/4 of the flask’s volume) into the flask. Heat the flask using an oil bath, maintaining a constant temperature of 90 °C for 10 h to ensure thorough modification of the nanocrystalline cellulose, resulting in highly lipophilic nanocrystalline cellulose. Take 3 g of the prepared highly lipophilic nanocrystalline cellulose and add it to 200 mL of anhydrous ethanol. Stir at speed of 300 r/min for 20 min, followed by ultrasonic dispersion at room temperature for 30 min to achieve a uniformly mixed and well-dispersed suspension system. Transfer the above suspension system into a 250 mL three-neck flask and slowly add 2 g of an organic amine surfactant dropwise. Continue stirring for 5 h. After the reaction, wash the product three times with anhydrous ethanol and vacuum-dry at 50 °C for 12 h to obtain pH-responsive nanocrystalline cellulose. Characterize the nanocrystalline cellulose before and after modification using electron microscopy to evaluate changes in its configuration and surface element distribution and analyze the differences between the modified and unmodified nanocrystalline cellulose. The electron microscope images were acquired with Hitachi SU8600 (5 kV, Au sputtering) (Hitachi High-Technologies Corporation, Tokyo, Japan). The EDS stratified image for NCC and MNCC were acquired with Hitachi SU8600 (15 kV, 60 s).

### 3.3. Preparation of the Initial Emulsion

The initial emulsion was prepared using a conventional method. An amount of 1.6 g of MNCC-type emulsifier was mixed with 100 mL of Scalan 5# white oil and stirred at 12,000 rpm for 5 min. Then, 100 mL of deionized water was added, followed by homogenization at 12,000 rpm for 40 min to form the initial emulsion (labelled as Type I emulsion).

### 3.4. Investigation of Reversible Emulsion Phase Inversion

For the acid-induced phase inversion, parallel experiments were conducted with different groups, each containing 100 mL of Type I emulsion. Different volumes of 5 wt% HCl solution were added to each group, followed by stirring at 12,000 rpm for 40 min. Then, the minimum volume of HCl required to induce a stable phase inversion was recorded. The resulting inverted emulsion was labelled as Type II emulsion.

For alkali-induced phase inversion (after acid-induced inversion), parallel experiments were performed using 100 mL of Type II emulsion from each group. Different volumes of 5 wt% NaOH solution were added, followed by stirring at 12,000 rpm for 40 min. Then, the minimum volume of NaOH required to induce a stable phase inversion was recorded. The resulting inverted emulsion was labelled as Type III emulsion.

Type I is the original water-in-oil emulsion. On the basis of Type I emulsion, Type II emulsion is formed after acid-induced phase inversion, which presents an oil-in-water state. Further, Type III emulsion is obtained by adding alkali to Type II emulsion to achieve re-phase inversion and restore the water-in-oil structure.

## 4. Conclusions

In summary, we established modified nanocrystalline cellulose as a highly effective, sustainable emulsifier for reversible emulsion drilling fluids. We characterized MNCC and assessed the performance. Structural analysis confirmed the successful surface modification of nanocrystalline cellulose, evidenced by significantly increased nitrogen content and superior dispersion. This tailored surface structure enables MNCC to stabilize reversible emulsions effectively and achieved remarkable performance with 48 consecutive reversible phase inversion cycles. Organic clay enhanced the emulsion stability when combined with MNCC, forming a composite interfacial film at the oil–water interface. The stability of both W/O and O/W emulsions increased proportionally with organic clay content due to the superior strength of this composite film. Crucially, we determined that organic clay addition must be maintained ≤2.50 g/100 mL to preserve reversible phase behavior. The inherent lipophilicity of organoclay dictates the phase inversion requirements: higher organic clay content necessitates increased acid dosage for W/O → O/W inversion but reduces alkali requirement for O/W → W/O inversion. The concentration of CaCl_2_ in the aqueous phase profoundly impacts reversible phase inversion and stability. Higher CaCl_2_ concentrations reduce the acid requirement for W/O → O/W inversion but increase the alkali requirement for O/W → W/O inversion. Furthermore, the stability of both emulsion types (O/W and W/O) diminishes as CaCl_2_ concentration rises. These effects arise from the dual action of CaCl_2_: (i) compression of the electrical double layer, altering the interfacial composition and interaction energies, and (ii) modification of the density difference between the aqueous and oil phases. These mechanisms act synergistically to govern the reversible inversion behavior. These findings provide critical insights for designing high-performance reversible emulsion drilling fluids. The identified optimal ranges for organoclay dosage and CaCl_2_ concentration, coupled with the understanding of their mechanistic roles and the proven efficacy of eco-friendly MNCC, offer practical guidelines for formulation optimization. This work advances the broader application potential of reversible emulsion technology in challenging shale oil and gas development environments.

## Figures and Tables

**Table 1:** Effect of organic clay on the reversible phase performance of emulsion.

Dosage of Organic Clay	Initial W/O Emulsion Preparation	Acid-Induced Phase Reversal	Alkali Induced Phase Reversal
0 g/100 mL	Yes	Achievable	Achievable
1.0 g/100 mL	Yes	Achievable	Achievable
2.0 g/100 mL	Yes	Achievable	Achievable
2.5 g/100 mL	Yes	Achievable	Achievable
3.0 g/100 mL	Yes	Not achievable	-

**Table 2:** pH of acid and alkaline dispersion systems with different dosages of organic clay.

Dosage of Organic Clay	HCl 1	HCl 2	HCl 3	NaOH 1	NaOH 2	NaOH 3
0 g/100 mL	6.5	4.6	3.3	8.7	9.2	11.1
1.0 g/100 mL	6.5	4.5	3.2	8.6	9.1	11.2
2.0 g/100 mL	6.4	4.6	3.3	8.8	9.2	11.1
2.5 g/100 mL	6.5	4.6	3.2	8.7	9.1	11.1

**Table 3:** Reversible phase inversion performance of emulsions prepared with different aqueous phase CaCl_2_ concentrations.

Concentration of CaCl_2_ in Water Phase	Feasibility of Preparing Initial W/O Emulsion	Feasibility of Acid-Induced Phase Inversion	Feasibility of Alkali-Induced Phase Inversion
0 wt%	Yes	Achievable	Achievable
15 wt%	Yes	Achievable	Achievable
30 wt%	Yes	Achievable	Achievable

**Table 4:** Performance indexes of acid-induced phase-inverted W/O emulsions prepared with different aqueous CaCl_2_ concentrations.

Concentration of CaCl_2_ in Water Phase	Demulsification Voltage (V)	Conductivity (µs cm^−1^)	Oil Separation Rate (24 h, Static)	Water Separation Rate (24 h, Static)
0 wt%	441	0	2%	0
15 wt%	289	0	10%	7%
30 wt%	204	0	13%	8%

**Table 5:** Performance indexes of alkali-induced phase-inverted water-in-oil emulsions prepared with different aqueous CaCl_2_ concentrations.

Concentration of CaCl_2_ in Water Phase	Demulsification Voltage (V)	Conductivity (µs cm^−1^)	Oil Separation Rate (24 h, Static)	Water Separation Rate (24 h, Static)
0 wt%	0	144	0	0
15 wt%	0	11,100	0	4%
30 wt%	0	11,100	1%	6%

**Table 6:** Acid- and alkali-induced phase transitions vs. CaCl_2_ concentration.

Type of Phase Transition Point	0% CaCl_2_	15% CaCl_2_	30% CaCl_2_
Acid turning point (5% HCl)	0.35%	0.28%	0.20%
Alkali-induced transition point (5% NaOH)	0.87%	0.95%	1.05%

## Data Availability

The original contributions presented in this study are included in the article. Further inquiries can be directed to the corresponding author.

## References

[1] Sun J., Jiang G., He Y., Shi H., Du M., Dong T., Yang L. (2023). Technical difficulties and challenges faced by oil-based drilling fluid. J. China Univ. Pet. Ed. Nat. Sci..

[2] Lyu K., Du H., Sun J. (2023). Research and development of oily drilling cuttings treatment technologies. J. China Univ. Pet. (Ed. Nat. Sci.).

[3] Yang S., Liu C., Le Nir I., Zhang T., Bloemenkamp R., Comparon L. Drilling risk migration based on borehole failure mechanism analysis from oil-base mud images: A case study from west of Shetland, North Sea. Proceedings of the SPE Europec Featured at EAGE Conference and Exhibition.

[4] Addagalla A.K., Kosandar B.A., Lawal I., Jadhav P.B., Yadav P. Using mesophase technology to remove and destroy the oil-based mud filter cake in wellbore remediation applications-case histories, Saudi Arabia. Proceedings of the SPE Middle East Oil and Gas Show and Conference.

[5] Ren Y., Lu Y., Jiang G., Zhou W., Wu L., Yao R., Xie S. (2021). Carbon dioxide/calcium oxide responsive behavior and application potential of amine emulsion. Pet. Explor. Dev..

[6] Santos J.M., Petri I.J., Mota A.C.S., dos Santos Morais A., Ataíde C.H. (2018). Optimization of the batch decontamination process of drill cuttings by microwave heating. J. Pet. Sci. Eng..

[7] Kogbara R.B., Ayotamuno J.M., Onuomah I., Ehio V., Damka T.D. (2016). Stabilisation/solidification and bioaugmentation treatment of petroleum drill cuttings. Appl. Geochem..

[8] Luyster M.R., Patel A.D., Ali S.A. Development of a delayed-chelating cleanup technique for openhole gravel-pack horizontal completions using a reversible invert emulsion drill-in system. Proceedings of the SPE International Conference and Exhibition on Formation Damage Control.

[9] Manzoleloua C., Nguyen C., Okhrimenko A., Traboulay V., Gamargo M., Li D. Deepwater Gas Injector Wells: Overcoming the Challenge of Achieving Matrix Injectivity. Proceedings of the SPE International Conference and Exhibition on Formation Damage Control.

[10] Eyaa Allogo C.-M., Allias B., Tayebi R., Baraque A., Lansot J.Y., Diogo J. Reversible fluids and mud breaker technology outperforms productivity and injectivity in west Africa–fit to purpose and designed for success. Proceedings of the SPE International Conference and Exhibition on Formation Damage Control.

[11] Lagaly G., Reese M., Abend S. (1999). Smectites as colloidal stabilizers of emulsions: I. Preparation and properties of emulsions with smectites and nonionic surfactants. Appl. Clay Sci..

[12] Nonomura Y., Kobayashi N. (2009). Phase inversion of the Pickering emulsions stabilized by plate-shaped clay particles. J. Colloid Interface Sci..

[13] Vignati E., Piazza R., Lockhart T.P. (2003). Pickering emulsions: Interfacial tension, colloidal layer morphology, and trapped-particle motion. Langmuir.

[14] Wilde P., Mackie A., Husband F., Gunning P., Morris V. (2004). Proteins and emulsifiers at liquid interfaces. Adv. Colloid Interface Sci..

[15] Asekomhe S.O., Chiang R., Masliyah J.H., Elliott J.A. (2005). Some observations on the contraction behavior of a W/O drop with attached solids. Ind. Eng. Chem. Res..

[16] Hsu M.F., Nikolaides M.G., Dinsmore A.D., Bausch A.R., Gordon V.D., Chen X., Hutchinson J.W., Weitz D.A., Marquez M. (2005). Self-assembled shells composed of colloidal particles: Fabrication and characterization. Langmuir.

[17] Pichot R., Spyropoulos F., Norton I. (2010). O/W emulsions stabilised by both low molecular weight surfactants and colloidal particles: The effect of surfactant type and concentration. J. Colloid Interface Sci..

[18] Liu F., Wang Y.L. (2017). Synthesis and performance study of the nanomaterial used to stabilize the reversible invert emulsion drilling fluid. Key Eng. Mater..

[19] Mariano M., El Kissi N., Dufresne A. (2014). Cellulose nanocrystals and related nanocomposites: Review of some properties and challenges. J. Polym. Sci. Part B Polym. Phys..

[20] Eyley S., Thielemans W. (2014). Surface modification of cellulose nanocrystals. Nanoscale.

[21] Grishkewich N., Mohammed N., Tang J., Tam K.C. (2017). Recent advances in the application of cellulose nanocrystals. Curr. Opin. Colloid Interface Sci..

[22] Sadeghifar H., Filpponen I., Clarke S.P., Brougham D.F., Argyropoulos D.S. (2011). Production of cellulose nanocrystals using hydrobromic acid and click reactions on their surface. J. Mater. Sci..

[23] Shen Y.-H. (2004). Phenol sorption by organoclays having different charge characteristics. Colloids Surf. A Physicochem. Eng. Asp..

[24] Park Y., Ayoko G.A., Frost R.L. (2011). Application of organoclays for the adsorption of recalcitrant organic molecules from aqueous media. J. Colloid Interface Sci..

[25] He H., Frost R.L., Bostrom T., Yuan P., Duong L., Yang D., Xi Y., Kloprogge J.T. (2006). Changes in the morphology of organoclays with HDTMA+ surfactant loading. Appl. Clay Sci..

[26] Taleb K., Abbou I., Raho R., Saidi-Besbes S. (2026). Comparative study of the stabilizing behavior of organoclays in Pickering emulsion. J. Dispers. Sci. Technol..

[27] Liu X., Li M.-C., Lv K., Sun J., Zhang Y., Liu C., Mei C. (2025). Inverse Pickering emulsion stabilized by modified cellulose nanocrystals for drilling fluid application. Colloids Surf. A Physicochem. Eng. Asp..

[28] Long H., Chen W., Tan D., Yang L., Zhang S., Wang S. (2021). Development of a high temperature and high pressure oil-based drilling fluid emulsion stability tester. Open J. Yangtze Oil Gas.

[29] Yan J., Wang F., Fan W., Su C. (1995). The emulsive stability of fine solid particles in oilewater systems. Oilfield Chem..

[30] Li J., Li Q., Li N., Teng X., Ren L., Liu H., Guo B., Li S., Hisham N.-E.-D., Al-Mujalhem M. Ultra-high density oil-based drilling fluids laboratory evaluation and applications in ultra-HPhT reservoir. Proceedings of the SPE Asia Pacific Oil and Gas Conference and Exhibition.

[31] Huang W., Lan Q., Qiu Z., Zhang Y., Zhong H., Feng G. (2016). Colloidal properties and clay inhibition of sodium silicate in solution and montmorillonite suspension. Silicon.

[32] Zhou J., Nasr-El-Din H. A new application of potassium nitrate as an environmentally friendly clay stabilizer in water-based drilling fluids. Proceedings of the SPE International Conference on Oilfield Chemistry.

[33] Luo Z., Wang L., Yu P., Chen Z. (2017). Experimental study on the application of an ionic liquid as a shale inhibitor and inhibitive mechanism. Appl. Clay Sci..

[34] Miller L., Witt J. (2002). Solubility of calcium hydroxide. J. Phys. Chem..

[35] Iwasaki S., Kodani S., Zushi Y., Hotta M., Hara M., Tatsuoka T., Koga N. (2023). Dissolution of calcium hydroxide in water: A guided inquiry in university and high school chemistry laboratories. J. Chem. Educ..

[36] Yuan G., Zhang Y., Dai H., Ye C., Wang Z. (2011). Effects of calcium and sodium ions on the Zeta potential of pulp. J. Nanjing For. Univ..

[37] Xu M., Li M., Peng B., Wu Z., Lin M., Guo J., Dong Z. (2007). Effects of strength of interfacial film and zeta potential on O/Wemulsion stability. Chin. J. Appl. Chem..

[38] Meng W., Quan-Fu A., Li-Guang W., Jian-Xiong M., Cong-Jie G. (2007). Recent developments in the measurement of zeta-potential of membrane. Chin. J. Anal. Chem..

